# Integrating LGBTQ Aging Related Content Into Post-Secondary Curricula

**DOI:** 10.1093/geroni/igaf122.1790

**Published:** 2025-12-31

**Authors:** Rajean Moone

**Affiliations:** University of Minnesota, Minneapolis, Minnesota, United States

## Abstract

Everyone deserves access to high-quality, compassionate care as they age. However, many LGBTQ older adults have faced discrimination when seeking services and often experience greater health, economic, and social disparities compared to their peers. With smaller informal support networks, they are more likely to rely on formal services—yet fear and past discrimination can discourage them from doing so. It is essential for students, especially those in healthcare-related fields, to understand the unique needs and challenges of LGBTQ aging. Despite this, few courses address the topic, and many faculty members have not been exposed to LGBTQ aging in their academic training. To bridge this gap, the Minnesota Northstar Geriatric Workforce Enhancement Program (GWEP) developed two resources: the LGBTQ Aging Care Toolkit (2023) and the HIV & Aging Toolkit (2024). These community-developed, peer-reviewed toolkits provide ready-to-use interprofessional modules to support faculty in integrating LGBTQ aging content into their curricula. This session will explore the development of these toolkits and strategies for incorporating them into existing aging-related courses.

